# Machine Learning Assisted Real-Time Label-Free SERS Diagnoses of Malignant Pleural Effusion due to Lung Cancer

**DOI:** 10.3390/bios12110940

**Published:** 2022-10-28

**Authors:** Jayakumar Perumal, Pyng Lee, Kapil Dev, Hann Qian Lim, U. S. Dinish, Malini Olivo

**Affiliations:** 1 Translational Biophotonics Laboratory, Institute of Bioengineering and Bioimaging, Agency for Science, Technology and Research (A*STAR), Singapore 138667, Singapore; 2 Respiratory and Critical Care Medicine, National University Hospital, Yong Loo Lin School of Medicine, National University of Singapore, Singapore 117597, Singapore

**Keywords:** lung cancer, pleural effusion, surface-enhanced Raman spectroscopy, clinical study, chemometrics, diagnosis

## Abstract

More than half of all pleural effusions are due to malignancy of which lung cancer is the main cause. Pleural effusions can complicate the course of pneumonia, pulmonary tuberculosis, or underlying systemic disease. We explore the application of label-free surface-enhanced Raman spectroscopy (SERS) as a point of care (POC) diagnostic tool to identify if pleural effusions are due to lung cancer or to other causes (controls). Lung cancer samples showed specific SERS spectral signatures such as the position and intensity of the Raman band in different wave number region using a novel silver coated silicon nanopillar (SCSNP) as a SERS substrate. We report a classification accuracy of 85% along with a sensitivity and specificity of 87% and 83%, respectively, for the detection of lung cancer over control pleural fluid samples with a receiver operating characteristics (ROC) area under curve value of 0.93 using a PLS-DA binary classifier to distinguish between lung cancer over control subjects. We have also evaluated discriminative wavenumber bands responsible for the distinction between the two classes with the help of a variable importance in projection (VIP) score. We found that our label-free SERS platform was able to distinguish lung cancer from pleural effusions due to other causes (controls) with higher diagnostic accuracy.

## 1. Introduction

Cancer is one of the many diseases with a high mortality rates, and an increasing prevalence of cancer due to the increase in aging population and adoption of unhealthy lifestyles has led to immense economic strains on healthcare systems, especially in developing countries [1]. Lung cancer, in particular, was the most diagnosed cancer, accounting for 13% of total cancer incidences and 20% of total cancer mortality as of 2012 [2]. It remains a lethal disease with one of the lowest 5-year survival rates of 10–15%. Between 1975 and 2009, patients diagnosed with clinical stage IV lung cancer had a 5-year overall survival rate of only 2% while those diagnosed with the lowest stage IA had a 5-year overall survival rate of 50% [3,4].

The typical diagnosing procedure includes a series of assessments, such as physical examination, imaging tests, and biopsies which can all be invasive and time consuming [5]. As with any disease, the need for accurate, rapid and non-invasive diagnostic methods for the early detection of lung cancer is crucial to good patient prognosis. Diagnosing and screening diseases using Raman spectroscopy is emerging as a promising viable avenue for cancer diagnosis as it allows for the spectral analysis of biofluids and tissues in the body. Several studies have attempted using this technique to identify biomolecular and chemical changes in biofluids, such as serum, plasma, saliva, cervical fluid, and urine in relation to cancer [6,7,8,9,10].

A study consisting of approximately 400 subjects with different types of cancer conditions has successfully differentiated normal patients from cancer patients with a sensitivity of 92% and specificity of 81% using a Raman spectral analysis of the serum collected. However, additional reports on the effectiveness of this method in investigating certain types of cancers have been limited. This is because Raman signals are intrinsically weak, so the Raman tool by itself is not sufficiently sensitive to warrant clinical value. Raman signal intensity can be enhanced by as much as an order of 10 by a phenomenon called surface-enhanced Raman spectroscopy (SERS) owing to different substrates [11,12,13]. SERS substrates can be fabricated by assembling metallic nanocolloidal clusters and nanoparticles with SERS properties onto surfaces of various shapes, or by an etching process to obtain planar nanostructures such as nanopillars [14,15]. Raman enhancement using such fabricated SERS substrates was successfully implemented in recent studies investigating Raman spectroscopy of biofluids [15,16]. Studies attempting biofluid SERS analysis have observed differences in Raman peaks and peak intensities between normal and diseased patients, allowing for a non-invasive diagnosis of oral, breast, head, neck, and cervical cancer [16,17,18].

In this pilot study, we explore the application of label-free SERS for the biochemical analysis of pleural fluid samples from control and cancer patients. We also investigate the potential of obtaining valuable information from the cancer patient samples compared with that of the control samples without having to target any specific biomarker or protein. Cancer is typically identified by observing and comparing the differences of spectrum and spectral parameters (peak position, peak height, peak area, and peak shape, etc.) between control samples and cancer patient samples. However, due to the limitation, complexity, and subjectivity of this traditional method, it is hard to obtain objective and accurate results. To extract meaningful information and systematically research the spectral data acquired, the method of chemical information, or chemometrics, is helpful and necessary. Herein, we have implemented a reliable and high level of uniformity across the SERS substrate to generate SERS spectra [14]. We have collected multiple SERS spectra via mapping for each pleural fluid sample. Controls and cancer patients were distinguished by using the SCSNP SERS substrate and pattern recognition methods of chemometrics, including principal component analysis (PCA), linear correlation analysis (LDA), partial least squares-discriminant analysis (PLS-DA), and uncorrelated linear discriminant analysis (ULDA). Our proof-of-concept study with a small sample size affirms the potential of the SERS platform in combination with the use of a binary classifier algorithm for the rapid diagnosis of lung cancer using pleural fluid with a high level of sensitivity and specificity.

## 2. Materials and Methods

Pleural fluid samples were transferred into 1.5 mL Eppendorf™ tubes and centrifuged at 10,000 rpm for 10 min at 4 °C to separate fluid from the cells. The supernatant was removed, aliquoted, and stored at −20 °C. Only samples with no signs of blood contamination were used for measurement.

**Figure 1:**
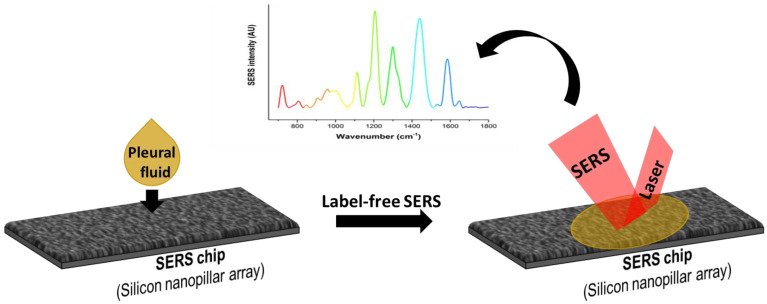
Schematic showing the label-free SERS platform for pleural fluid analysis.

Prior to use, these substrates were cleaned with ethanol followed by Millipore water to increase the wettability of aqueous samples. Stored pleural fluid was thawed and 5 μL placed onto the substrate. SERS measurements of the pleural fluid were carried out using a Renishaw InVia Raman upright microscope (Renishaw InVia, Gloucestershire, UK) with a 785 nm laser. A microscope (Leica) was integrated with this Raman system and the laser light was coupled through an objective lens (50X, 0.75 N.A), which was used to excite the sample and to collect the scattered Raman signal. The dominant Rayleigh scattering was blocked using a notch filter and the beam spot on the sample was ~1 µm. A minimum of 500 spectra per pleural fluid sample were acquired via mapping over 3 different locations. Integration time for each spectrum was 10 sec and the resultant mapping data were collected in the range of 700–1800 cm^−1^ wavenumber region. Figure 1 shows the schematic for the label-free SERS measurement of the pleural fluid on an SCSNP substrate. The fabrication of the SCSNP has been described in our earlier work [14]. Briefly, an etching method based on blanket inductive coupled plasma (ICP) was used to etch the Si wafer to generate a random silicon-based nanograss structure followed by pure silver (Ag) vapour deposition using the E-beam evaporation process.

In this pilot study, we acquired the SERS spectra from pleural fluid samples of subjects with different medical conditions (lung, breast, ovarian cancer, etc.) using benchtop confocal Raman spectroscopy. The Raman fingerprint for the pleural fluid sample was acquired between 735 cm^−1^ to 1700 cm^−1^ and was further used in the chemometrics analysis. Multiple spectra were acquired for each subject via mapping the related pleural fluid SERS sample. The mapping data in terms of multiple SERS spectra were affected by autofluorescence from the sample when illuminated with the laser light source in the confocal Raman spectroscopy system. A representative pleural fluid SERS spectra obtained using ~220 individual spectra across multiple SERS substrates is shown in Figure S1 (Supplementary Material). The autofluorescence from the sample overlapped with the SERS spectral signal from the sample under investigation and, hence, caused a deviation from the linear relationship between the SERS signal intensity and the associated molecular concentration [19,20]. Thus, it was important to preprocess the SERS spectra and remove any autofluorescence baseline effect. In order to subtract the effect of autofluorescence mathematically, a spectral baseline correction was implemented using the asymmetric least squares (AsLS) baseline correction method [21]. The baseline-corrected SERS spectra were further processed using the Savitzky–Golay smoothing algorithm in order to remove any contribution from the cosmic ray spikes and enhance the signal-to-noise ratio (SNR) [22]. The preprocess routine was repeated for each SERS spectrum acquired for each pleural fluid sample. The baseline-corrected SERS spectrum with improved SNR from the subject was further subdivided between the control and cancer groups and analyzed using the partial least squares-discriminant analysis (PLS-DA) binary classification method.

We implemented a supervised PLS-DA based on the partial least squares regression (PLSR) for dimensionality reduction and further classification. The PLS regression-based PLS-DA binary classification method further rotated the components (latent variables (LVs)) to achieve the maximum group separation compared with the PCA model resulting into better classification results. The basic theory behind the PLS-DA classification method has been described elsewhere [23,24,25]. To validate the accuracy of this PLS-DA binary classification model, a stratified K (10)-fold cross-validation method was used that was helpful to further reduce classification errors resulting from class imbalance. The variable importance in projection (VIP) score is an important parameter that can be evaluated from the PLS-DA classification model and estimates the importance of each variable. The VIP score is the weighted sum correlation between PLS latent variables. Variables with a VIP score value greater than the numerical value of one (1) are considered as important and can further help to optimize the PLS-DA classification model [26]. The VIP score helps to identify important wavenumbers or Raman band regions that are significantly different in two groups under investigation, i.e., the VIP score can be further used to discriminate between the two subject groups by selecting certain wavenumbers.

In this study, we investigated pleural fluid samples from three different classes, i.e., lung cancer, all other conditions of cancer, and control subjects. To understand how the pleural fluid SERS signature could assist to identify the different health condition, we used the advanced machine learning tools noted above.

## 3. Results and Discussion

**Figure 2:**
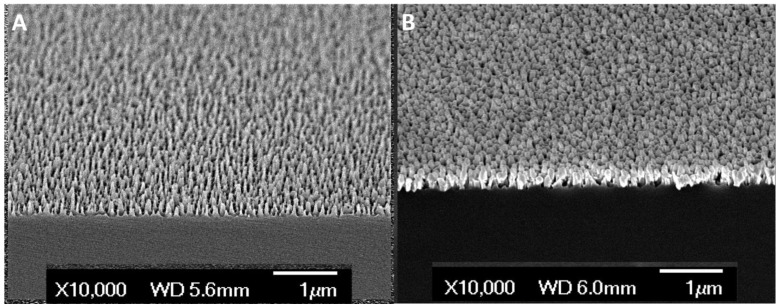
FE-SEM cross-sectional view of silicon nanopillar (SNP) structure. (**A**) Clean SNP without metal coating. (**B**) Silver coated silicon nanopillar (SCSNP).

SCSNP substrates were fabricated as described in the materials and methods section. For SERS measurements on the pleural fluid samples, a silver coated silicon nanopillar (SCSNP) was fabricated in-house. Figure 2A,B shows the silicon nanopillar (SNP) before and after silver coating, respectively [14]. We had also tested with a commercial SERS substrate from Silmeco (Denmark) for the initial studies.

Excess pleural fluid is secreted when the pulmonary system is triggered by irritation or inflammation that could be due to infections like tuberculosis (TB), allergies, or from various cancers. When it comes to cancer totals, the major contributors are lung cancer or breast cancer. In our study we collected pleural fluid samples from a total of 34 patients; we have provided the details of the patients and their condition in Table 1. The breakdown based on the type of cancer and the non-cancer subjects are also shown in Table 1. Briefly, there were 15 patients with lung cancer, 7 with other cancers, and 12 controls (who were non-cancerous and had pleural fluid secretions due other infections).

### 3.1. Case One: Lung Cancer vs. Control

**Figure 3:**
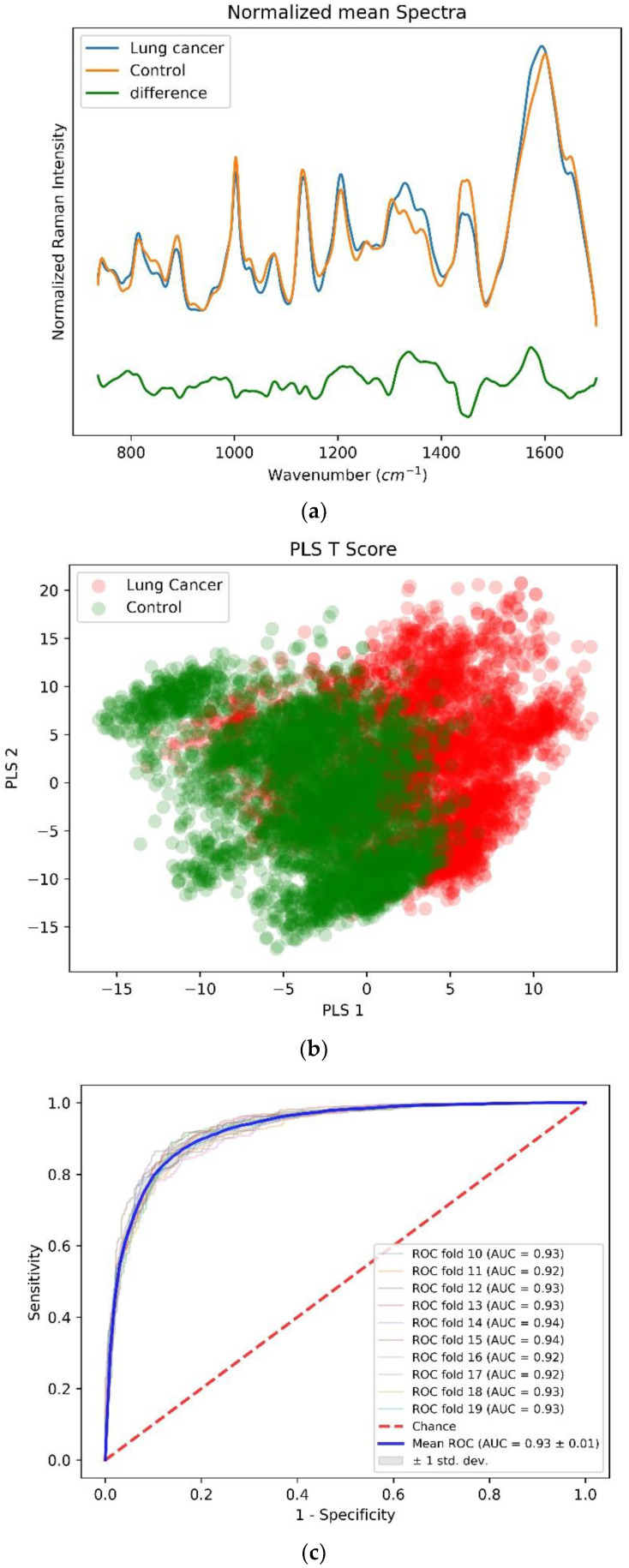

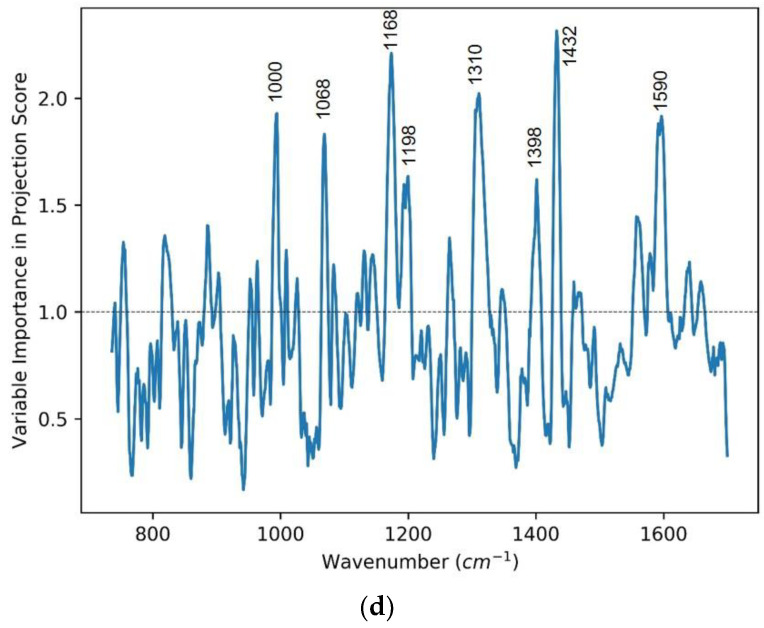
(**a**) Mean-centered, preprocessed mean SERS spectra and their difference for lung cancer (n = 12) and control (n = 11) groups within a wavenumber range of 735–1700 cm^−1^. (**b**) Two-dimensional scatterplot for first two latent LVs from the PLS-DA classification model. (**c**) Receiver operating characteristics (ROC) curve demonstrating classification capability of PLS-DA classification model at different thresholds. (**d**) Variable importance in projection (VIP) score highlighting important discriminative wavenumber bands between lung cancer and control group.

Figure 3a shows that for the mean SERS spectra for the lung cancer (n = 12 subjects) and control (n = 11 subjects) groups, each group constituted approximately 4000 SERS spectra. A great difference between the lung cancer and control subjects mean normalized SERS spectrum is observed in the spectral ranges of 1000–1100 cm^−1^, 1200–1400 cm^−1^, 1440 cm^−1^, and 1500–1600 cm^−1^. These spectral ranges are related to protein (Amide III and Amide II) and lipid conformations (CH2 scissoring vibration). Zhiwei et al. distinguished tumor tissue from normal bronchial tissue with a sensitivity and specificity of 94% and 92%, respectively, using near infrared Raman spectroscopy [27]. The spectral difference achieved on the bronchial tissue specimen are consistent with our observation from the pleural effusion on the SERS platform. Thus, this method could be preferred to distinguish lung cancer subjects from control subjects by using the non-invasive pleural fluid method on the SERS platform.

The mean-centered, baseline-corrected SERS spectra within the 735–1700 cm^−1^ fingerprint wavenumber range from the two study groups are used as the descriptor (X) matrix, whereas the response (Y) vector has been artificially generated to designate group affinities. PLS-DA determines the fit between the descriptor matrix and class groups by maximizing the covariance and, as a result, latent variables (LVs) in terms of the PLS score are determined. Figure 3b shows the scatterplot of the first two LVs from the PLS score showcasing the group clustering from each subject group. To validate the accuracy of this PLS-DA classification model, a stratified K (10)-fold cross-validation method is used, which preserves the proportions of subgroups under training and test samples. A classification accuracy of 0.85 is achieved along with a sensitivity and specificity of 87% and 83%, respectively. Figure 3c shows an averaged receiver operating characteristic (ROC) curve with mean value the area under curve of 0.93 from multiple cross-validation folds that demonstrates the capability of the PLS-DA binary classifier to distinguish between lung cancer and control subjects with threshold variation. Finally, Figure 3d shows the VIP score evaluated from the PLS-DA classification model. The VIP score with a numerical value greater than one (1) indicates that the spectral bands and wavenumbers are discriminative between the two subject groups. The most discriminative (higher VIP score or width of the band) wavenumber bands, their peak positions, and the vibrational mode assignment have been explained in Table 2 below. As evident from the spectral difference between the two subject groups, the VIP score also shows that the protein and lipid conformations are the most discriminative.

From the VIP score that is evaluated using the PLS-DA classification model, the 1002 cm^−1^, 1068 cm^−1^, 1160–1205 cm^−1^, 1300–1330 cm^−1^, 1420–1440 cm^−1^, 1558 cm^−1^ and 1580–1600 cm^−1^ wavenumbers and wavebands constitute a scoring value of greater than one (1). This signifies the role of these wavenumbers and wavebands as discriminative between the two subject groups under the PLS-DA classification model. Although the mean normalized difference spectrum shows a great difference between the two subject groups’ mean spectra at multiple spectral locations, the VIP score from the PLS-DA classification model evaluates the most discriminative wavenumbers, waveband peak positions, and the related vibrational mode assignment as tabulated in Table 2. In alignment with other published research, the SERS-based lung cancer pleural fluid shows the spectral difference with respect to control subjects at the major protein and lipid conformations as depicted in Table 2.

### 3.2. Case 2: All Cancer vs. Control

**Figure 4:**
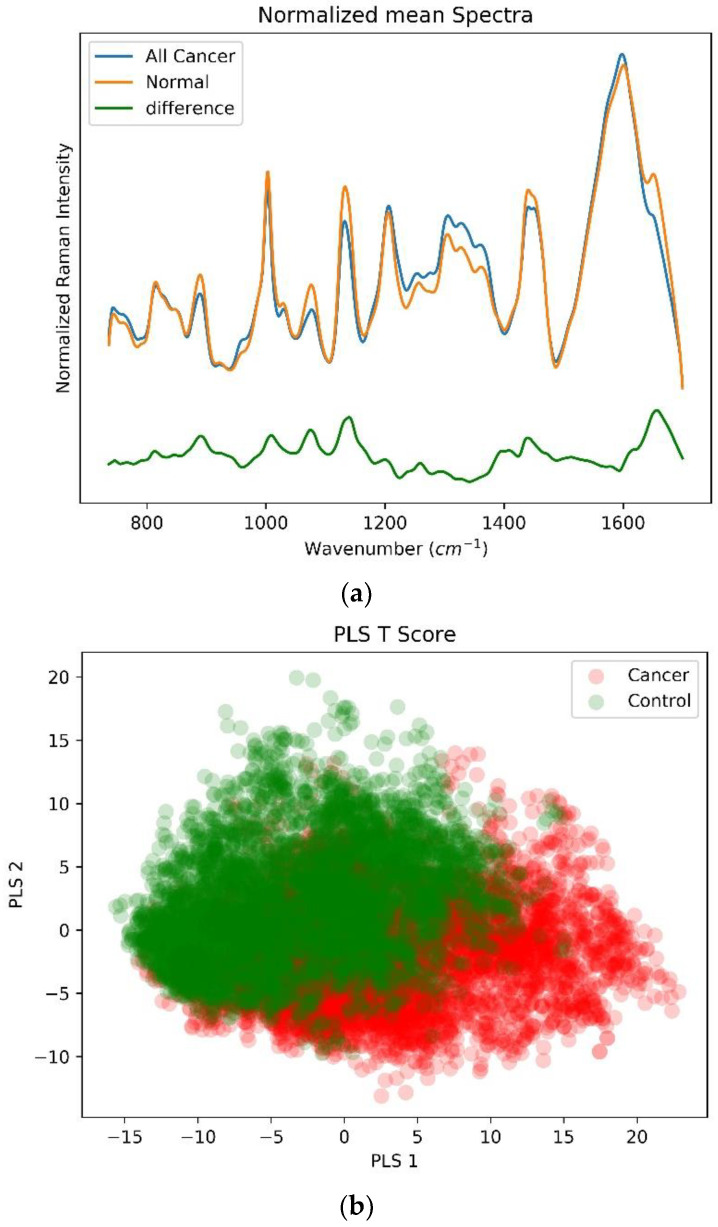

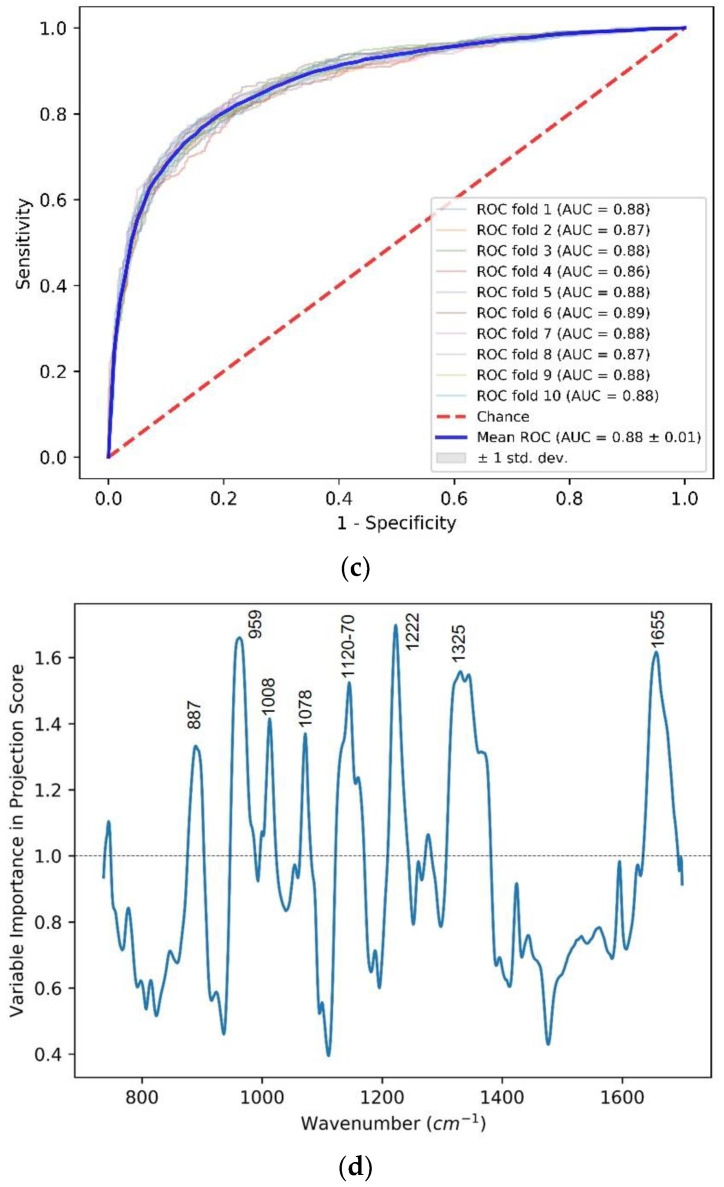
(**a**) Mean-centered, preprocessed mean Raman spectra and their differences for all cancer (n = 22) and control (n = 12) groups within a wavenumber range 735–1700 cm^−1^. (**b**) Two-dimensional Scatterplot for first two latent LVSs from the PLS-DA classification model. (**c**) Receiver operating curve (ROC) demonstrating classification capability of PLS-DA classification model at different thresholds. (**d**) Variable importance in projection (VIP) score highlighting important discriminative wavenumber bands between lung cancer and control groups.

The PLS-DA binary classification model is used to classify all cancers (n = 22, ~6000 Raman spectra’s) found in the subjects into the specific cancer types (15 lung, 1 lymphoma, 1 ovarian, 1 breast, 1 peritoneal, 2 malignant mesothelioma and 1 multiple myeloma) and control group (n = 12, ~5000 Raman spectra); please refer to Table 1 for more details. Figure 4a shows the mean-centered, preprocessed mean Raman spectra for these two subject groups along with their spectral difference value. Figure 4b shows the two-dimensional scatterplot using the first two LVs from the two subject groups evaluated using the PLS regression model. Here again, the spectral data are used as the descriptor, whereas the artificial affinities are defined for the two different subject groups for the supervised classification. In order to evaluate the accuracy of the PLS-DA classification model, a stratified K (10)-fold cross-validation method is used. For this PLS-DA classification model, a classification accuracy of 81% is achieved with the sensitivity and specificity of 77% and 84%, respectively, to predict the cancer condition. An ROC curve computed for all the cross-validation folds is shown in Figure 4c along with the VIP score in Figure 4d. The important discriminative wavenumber peaks and wavebands evaluated with the help of the VIP score are tabulated in Table 3 along with their respective peak positions and vibrational mode assignments [27,29,30].

This study was performed as a proof-of-concept demonstration to show the feasibility of using the label-free SERS technique in combination with the robust SERS substrate SCSNP. There are still some limitations such as the patient sample size not being substantial enough to offset the individual variation that might arise from different patients. We did not take into consideration the smoking or non-smoking aspect of the patients in this study. Moreover, the control patients’ pleural effusions may have more variations due to different infections. In our future work, we have planned to expand the study with a larger patient cohort that has a statistically significant number of lung cancer and control along with TB patients in order to be able to develop a more distinctive and robust algorithm that can, with the help of the SERS spectra, classify the pleural effusion as a cancer or TB.

## 4. Conclusions

This proof-of-concept study with a small sample size affirms the potential of the SERS platform in combination with the use of a binary classifier algorithm for the rapid diagnosis of lung cancer using pleural fluid. We have used chemometrics methods comprising principal component analysis followed by linear discrimination analysis (PCA-LDA) and partial least squares-discriminant analysis (PLS-DA), etc., in our current study to derive the disease condition. We report a classification accuracy of 85% along with a sensitivity and specificity of 87% and 83%, respectively, for the detection of lung cancer over control pleural fluid samples with an area under the ROC curve value of 0.93 using the PLS-DA binary classifier to distinguish between lung cancers over control subjects. We have also evaluated the discriminative wavenumber bands responsible for the differentiation between the two classes with the help of the variable importance in projection (VIP) score. Our proof-of-concept study demonstrates the feasibility of using the label-free SERS technique in combination with advanced chemometrics methods along with the robust and reproducible SERS substrate (SCSNP) to rapidly diagnose the presence of lung cancer using just the pleural effusions of the patients.

## Figures and Tables

**Table 1:** List of patients with lung cancer, other cancers, and control subjects.

Cancer Types	No. of Subjects
LUNG CANCER	15
OTHER CANCERS	
- BREAST CANCER	1
- OVARIAN CANCER	1
- PERITONEAL CANCER	1
- MALIGNANT MESOTHELIOMA	2
- MULTIPLE MYELOMA	1
- LYMPHOMA	1
NON-CANCER (CONTROL)	
- Exudate	8
- Transudate	4
TOTAL	34

**Table 2:** Assignment of Raman wavenumbers and bands evaluated using VIP score. ν = stretch, δ = deformation [28].

Peak Position (cm^−1^)	Vibrational Mode Assignment [26]
1002	Phenylalanine, C-C aromatic ring stretching
1068	C-C skeletal stretching (lipids)
1168	ν (C═C) δ(COH) (lipid)
1198	Nucleic acids and phosphates
1310	CH_3_/CH_2_ twisting or bending mode of lipid/protein assignment
1398	C═O symmetric stretch, CH_2_ deformation
1432	CH_2_ scissoring vibration (lipid)
1590	C═C olefinic stretch (protein)

**Table 3:** Assignment of Raman wavenumbers and bands evaluated using VIP score. ν = stretch, δ = deformation [28].

Peak Position (cm^−1^)	Vibrational Mode Assignment [26]
887	Protein bands, Structural protein modes of tumors
959	Single bond stretching vibrations for the amino acids proline, valine and polysaccharides
1008	Phenylalanine ν(CO), ν(CC), δ(OCH)
1078	ν(C-C) or ν(C-O), phospholipids and nucleic acid
1120–1170	ν(C-N), C-C skeletal trans conformation, phospholipidsνC-C of proteins (also carotenoids)
1222	Amide III (*β*-sheet)
1325	CH_3_CH_2_ wagging mode in purine bases of nucleic acids
1655	Amide I (protein) and C═C stretch (lipid)

## Data Availability

The data generated or analyzed during this study are included in the main article or supporting information.

## Supplementary Materials

The following supporting information can be downloaded at: https://www.mdpi.com/article/10.3390/bios12110940/s1, Figure S1: Overlay plot of representative pleural fluid SERS spectra obtained using ~220 individual spectra across multiple SERS substrates.
