# Supplementary material for: Machine Learning Assisted Real-Time Label-Free SERS Diagnoses of Malignant Pleural Effusion due to Lung Cancer
Source: Biosensors (Basel). 2022 Oct 28;12(11):940. doi: 10.3390/bios12110940 (PMC9688333; doi:10.3390/bios12110940)
Supplement: Supplementary file 1 [file biosensors-12-00940-s001.zip › biosensors-1911797-supplementary.pdf]

biosensors MDPI

Supplementary Material

Machine Learning Assisted Real-Time Label-Free SERS Diagnoses of Malignant Pleural Effusion due to Lung Cancer

Jayakumar Perumal [1], Pyng Lee [2,*,†], Kapil Dev [1,†], Hann Qian Lim [1], U. S. Dinish [1] and Malini Olivo [1,*]

1 Translational Biophotonics Laboratory, Institute of Bioengineering and Bioimaging, Agency for Science, Technology and Research (A*STAR), Singapore 138667, Singapore; jayakumar_perumal@ibb.a-star.edu.sg (J.P.); letstalkkapildev@gmail.com (K.D.); hannqian1818@hotmail.com (H.Q.L.); dinish@ibb.a-star.edu.sg (U.S.D.)

2 Respiratory and Critical Care Medicine, National University Hospital, Yong Loo Lin School of Medicine, National University of Singapore, Singapore 117597, Singapore

* Correspondence: mdclp@nus.edu.sg (P.L.); malini_olivo@ibb.a-star.edu.sg (M.O.); Tel.: +65-68247003 (M.O.)

† These authors contributed equally to this work.

We have provided a representative SERS spectra of patient pleural fluid obtained using our silver-coated silicon nanopillar (SCSNP) substrate that shows the repeatability of SERS signals from the pleural effusions. This plot shows the reliability of our SERS substrate to generate more reproducible SERS spectra across different regions of the multiple substrates.

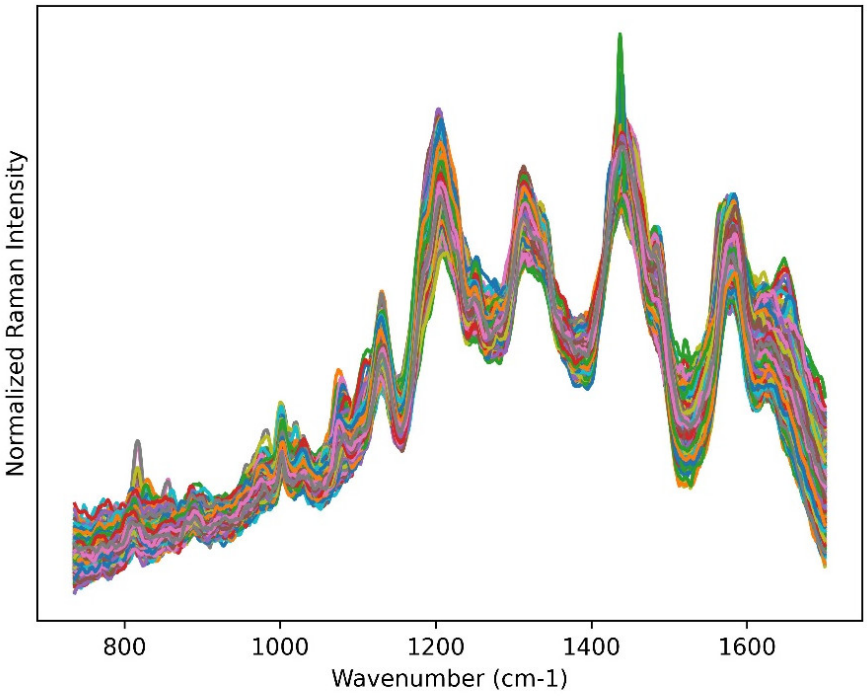

Figure S1. Overlay plot of representative pleural fluid SERS spectra obtained using ~220 individual spectra across multiple SERS substrates.
